# Ion Channel–Rho GTPase Coupling in Human Odontogenesis: Integrative Structural and Network Dissection of TRIO and CACNA2D2 Variants From Familial Tooth Agenesis

**DOI:** 10.7759/cureus.109624

**Published:** 2026-05-25

**Authors:** Fidele Nabbout, Joseph Sabbagh, Joelle EL Hajj, Michella Ghassibe

**Affiliations:** 1 Orthodontics, Faculty of Dental Medicine Lebanese University, Beirut, LBN; 2 Restorative Dentistry and Endodontics, Lebanese University, Beirut, LBN; 3 Molecular Biology, Saint Luc Hospital, Brussels, BEL; 4 Natural Sciences, School of Arts and Sciences, Lebanese American University, Beirut, LBN

**Keywords:** calcium signaling, in silico analysis, odontogenesis, rho gtpase signaling, tooth agenesis, voltage gated calcium channels

## Abstract

Introduction: Tooth agenesis is a genetically heterogeneous developmental anomaly in which disturbances of morphogen signaling, cytoskeletal organization, and mineralization converge during odontogenesis. Building on a previously published clinical and exome study of two Lebanese families with familial nonsyndromic tooth agenesis that identified rare segregating missense variants in trio Rho guanine nucleotide exchange factor (TRIO) and calcium voltage-gated channel auxiliary subunit alpha2delta 2 (CACNA2D2), this work examines how these genes may participate in a shared mechanistic axis during human tooth development.

Methods: We performed a secondary, exclusively in silico mechanistic analysis of the originally reported variants, TRIO c.8312C>T (p.Ser2771Leu) and CACNA2D2 c.284G>A (p.Arg95His), without enrolling new participants or generating additional sequencing or clinical data. Protein domain organization was annotated from curated resources, residue-level and local-window conservation were quantified across vertebrate orthologs, and the structural context of each substituted residue was inspected using available experimental templates and full-length AlphaFold models from the AlphaFold Protein Structure Database. Population allele frequencies and clinical annotations were re-evaluated in large reference datasets, and high-confidence protein-protein interaction networks centered on TRIO, ras homolog family member A (RHOA), guanine nucleotide binding protein subunit beta 1 (GNB1), and CACNA2D2 were constructed to assess whether these genes form a common odontogenesis-relevant signaling landscape.

Results: Both substitutions localize to structured and functionally annotated regions: CACNA2D2 Arg95 lies in the extracellular N-terminal segment immediately upstream of the von Willebrand factor type A (VWA) domain, and TRIO Ser2771 lies within an immunoglobulin I-set domain adjacent to a C-terminal kinase-like region. In both cases, the affected residue is highly conserved across mammalian orthologs and falls within a locally conserved window. TRIO p.Ser2771Leu remains absent from population reference datasets, whereas CACNA2D2 p.Arg95His (reference SNP identifier, rs149979955) is a very rare allele with a non-dental Clinical Variants (ClinVar) annotation; both variants receive predominantly deleterious predictions. Structural inspection suggests possible effects on local packing, electrostatics, or interaction interfaces. Network analysis places TRIO, RHOA, GNB1, and CACNA2D2 along a high-confidence path enriched for cytoskeletal regulation, vesicle trafficking, calcium signaling, and mineralization, consistent with an ion channel-Rho guanosine triphosphatase (GTPase) coupling model in odontogenesis.

Conclusions: This structural and network analysis suggests that the reported TRIO and CACNA2D2 variants may contribute to a testable TRIO-RHOA-GNB1-CACNA2D2 axis in human tooth development. The evidence remains hypothesis-generating and does not establish pathogenicity or causality, but it provides a coherent framework for targeted functional studies in dental and craniofacial models.

## Introduction

Tooth agenesis is among the most common developmental anomalies of the permanent dentition and reflects disruption of early tooth initiation, morphogenesis, and terminal differentiation programs [1-5]. Its genetic architecture is highly heterogeneous and encompasses both isolated and syndromic forms, with established contributions from canonical developmental pathways such as Wnt signaling (WNT), ectodysplasin A/ectodysplasin A receptor/nuclear factor kappa‑light‑chain‑enhancer of activated B cells (EDA/EDAR/NF‑κB), bone morphogenetic protein (BMP), fibroblast growth factor (FGF), Sonic hedgehog (SHH), and multiple transcription‑factor networks [1,2,4-7].

Beyond these classical pathways and recurrent tooth agenesis genes, normal odontogenesis also depends on dynamic cytoskeletal remodeling, epithelial-mesenchymal interactions, and tightly regulated calcium handling during enamel and dentin formation. These broader cellular processes imply that genes not traditionally represented on standard dental genetics panels may contribute to tooth agenesis when their perturbation affects morphogenesis, integration of signaling inputs, or matrix mineralization [1-3,6-8].

During human odontogenesis, epithelial-mesenchymal interactions progressively generate distinct tooth‑germ stages (bud, cap, and early and late bell), culminating in the differentiation of inner enamel epithelium into ameloblasts and dental papilla mesenchyme into odontoblasts, which are responsible for enamel and dentin secretion, respectively. Recent bulk and single‑cell transcriptomic surveys of human and murine tooth germs have mapped the signaling programs that guide these transitions, highlighting stage‑specific activation of WNT, BMP, FGF, transforming growth factor beta (TGFβ), and Hedgehog (HH) pathways in ameloblast and odontoblast lineages [1-3]. Within these datasets, cytoskeletal remodeling and calcium‑handling pathways are recurrently enriched as ameloblasts and odontoblasts acquire their polarized, matrix‑secreting phenotypes, supporting a biologically plausible interface between Ras homolog family member (Rho) guanosine triphosphatase (GTPase) signaling, vesicle trafficking, and voltage‑gated calcium‑channel function in tooth development [1-3,6,7]. In this context, the trio Rho guanine nucleotide exchange factor (TRIO)-ras homolog family member A (RHOA)-guanine nucleotide binding protein subunit beta 1 (GNB1)-calcium voltage‑gated channel auxiliary subunit alpha2delta 2 (CACNA2D2) axis proposed here is intended as a mechanistic framework for how ion channel-Rho GTPase coupling might contribute to morphogenesis and mineralization in odontogenic cell populations, rather than as a pathway restricted to generic cell biology [1-3,6,7,9].

Within this broader framework, Rho GTPase-dependent cytoskeletal regulators and auxiliary subunits of voltage‑gated calcium channels emerge as attractive mechanistic candidates in tooth development [1-3,6,7,9]. Guanine nucleotide exchange factors acting on Rho family members can influence cell migration, polarity, and tissue architecture in the craniofacial complex, whereas α2δ subunits of calcium channels modulate channel trafficking, membrane localization, and calcium influx in both excitable and non‑excitable cells [1-3,6,7].

A previously published clinical and whole‑exome sequencing study of two Lebanese families with familial nonsyndromic tooth agenesis identified rare segregating missense variants in TRIO, a multidomain Rho guanine nucleotide exchange factor, and CACNA2D2, an α2δ subunit of voltage‑gated calcium channels. That work established these variants as candidate contributors in an understudied population but did not delineate how TRIO‑mediated Rho GTPase signaling and CACNA2D2‑related calcium‑channel biology might converge within a shared odontogenic pathway.

Objectives

The present study, therefore, undertakes an exclusively in silico, mechanistic extension of those previously reported variants as a system-level analysis rather than a second discovery report. Our objectives were to (1) refine the protein‑domain context of the TRIO c.8312C>T (p.Ser2771Leu) and CACNA2D2 c.284G>A (p.Arg95His) substitutions, (2) quantify residue‑level and local‑window evolutionary conservation to assess positional constraint, (3) examine the structural environment of each variant using experimental and AlphaFold Protein Structure Database (AlphaFold)-based models, (4) re‑evaluate population allele frequencies and computational pathogenicity support in large reference and clinical datasets, and (5) construct high‑confidence interaction networks centered on TRIO, RHOA, GNB1, and CACNA2D2 to test whether these genes can be embedded within a shared signaling axis consistent with ion channel-Rho GTPase coupling in human odontogenesis.

## Materials and methods

Study design and data source

This study is an exclusively in silico, secondary analysis based on previously published clinical and genetic data from two Lebanese families with familial nonsyndromic tooth agenesis carrying segregating trio Rho guanine nucleotide exchange factor (TRIO) and calcium voltage‑gated channel auxiliary subunit alpha2delta 2 (CACNA2D2) missense variants. The Lebanese families, their clinical and radiographic phenotypes, and the TRIO c.8312C>T (p.Ser2771Leu) and CACNA2D2 c.284G>A (p.Arg95His; reference SNP identifier [rs] 149979955) variants were originally identified and characterized in a clinical and whole‑exome sequencing study of familial tooth agenesis in Lebanese patients [9]. Full details of recruitment, pedigree structure, clinical examinations, radiographic assessment, consent procedures, and exome workflows, including variant calling and segregation analysis, were reported in that original publication. In the present work, no new human participants were enrolled, no additional biological samples were collected, and no new sequencing or clinical data were generated; only the previously reported variant calls were used as starting points for further computational analysis.

The research involving human participants was conducted in accordance with institutional and international ethical standards. Approval was obtained from the Lebanese American University Institutional Review Board and Committee on Human Subjects in Research (approval number LAU.SAS.MS1; 23 May 2018), with all procedures conforming to the Declaration of Helsinki, and written informed consent was obtained from all adult participants and from parents or legal guardians of minors, with assent from children when appropriate.

Variants selected for mechanistic analysis

The variants selected for mechanistic evaluation were TRIO c.8312C>T (p.Ser2771Leu) in family 1 and CACNA2D2 c.284G>A (p.Arg95His; rs149979955) in family 2. These substitutions were prioritized because they had been shown to segregate with tooth agenesis in their respective families in the original study, occurred in genes with plausible biological relevance to craniofacial development and calcium‑dependent odontogenesis, and remained candidate variants after initial filtering and interpretation [1-3,6,7,9].

Protein domain annotation

Canonical transcript and protein identifiers for TRIO and CACNA2D2 were retrieved from the National Center for Biotechnology Information (NCBI) Gene and Ensembl on the GRCh37/hg19 assembly. Domain organization was annotated using the Universal Protein Resource (UniProt) and Protein Families Database (Pfam), with specific attention to immunoglobulin‑like I‑set domains, Rho guanine nucleotide exchange factor and pleckstrin homology modules, and the C‑terminal kinase‑like region in TRIO, as well as the signal peptide, extracellular N‑terminal segment, von Willebrand factor type A (VWA) domain, and transmembrane region in CACNA2D2. Variant positions were mapped to canonical protein sequences using Human Genome Variation Society (HGVS) nomenclature, and linear schematics were generated to illustrate domain context and the spatial relationship between variant sites and neighboring functional modules.

Multiple‑sequence alignment and conservation analysis

Ortholog protein sequences for TRIO and CACNA2D2 were collected for 20 vertebrate species, including 15 mammals and 5 non‑mammalian vertebrates, using NCBI Basic Local Alignment Search Tool (BLAST), HomoloGene, and orthology resources. Only sequences annotated as clear one‑to‑one orthologs and with adequate coverage across the variant‑containing regions were retained. Multiple‑sequence alignments were generated with the Constraint‑based Multiple Alignment Tool (COBALT) and visualized with the NCBI Multiple Sequence Alignment Viewer using amino‑acid property‑based coloring. For CACNA2D2, a local window spanning residues 83-127 was examined to encompass Arg95; for TRIO, a local window spanning residues 2750-2790 was used to encompass Ser2771. For each species, percent identity to the human reference sequence was calculated across both the local window and the full‑length protein to contextualize local conservation against global similarity. Residues were considered highly conserved when the human amino acid was preserved in all mammalian orthologs and retained in most non‑mammalian vertebrates included in the panel [1-3,6,7].

Structural context assessment

Experimentally determined structures encompassing relevant protein regions were reviewed from the Protein Data Bank (PDB) when available, and full-length AlphaFold Protein Structure Database (AlphaFold) models for human TRIO and CACNA2D2 were inspected to examine residue environment [10]. Variant sites were mapped onto these models using molecular visualization software (e.g., PyMOL molecular graphics system [PyMOL] or UCSF ChimeraX molecular visualization system [ChimeraX]) to assess approximate secondary‑structure context, predicted local confidence, relative burial versus solvent exposure, proximity to domain interfaces, and potential participation in interaction‑ or ligand‑sensitive surfaces [10]. Structural interpretation was qualitative; we did not perform free‑energy‑change (ΔΔG) calculations, explicit protein‑stability predictions, interface‑disruption modeling, or position‑specific conservation scoring with tools such as Conservation Surface Mapping (ConSurf), nor did we undertake molecular‑dynamics simulations [10].

Population and clinical database review

Allele frequencies for TRIO p.Ser2771Leu and CACNA2D2 p.Arg95His were re‑checked in population reference datasets, including Genome Aggregation Database (gnomAD), Trans‑Omics for Precision Medicine (TOPMed), and Population Architecture using Genomics and Epidemiology (PAGE), where available, together with Single Nucleotide Polymorphism Database (dbSNP) for rs149979955 [9,11,12]. Variant annotations were reviewed in Clinical Variants (ClinVar) and interpreted in light of their prior reported clinical context [11,12]. For the purposes of this study, variants were considered very rare when the maximum reported allele frequency remained below thresholds commonly used for rare dominantly inherited disease candidates and absent when not observed in gnomAD exome and genome datasets accessible at the time of review [9,11,12].

In silico prediction framework

The computational prediction framework followed and extended the previously reported analysis, compiling results from six established missense prediction tools: Sorting Intolerant From Tolerant (SIFT), Polymorphism Phenotyping version 2 (PolyPhen‑2), likelihood ratio test (LRT), MutationTaster, MutationAssessor, and Functional Analysis through Hidden Markov Models (FATHMM) [7,9,11,12]. Variants were considered to have supportive computational evidence for deleteriousness when at least three of six tools predicted damaging functional impact, and concepts from American College of Medical Genetics and Genomics/Association for Molecular Pathology (ACMG/AMP) variant-interpretation guidelines (e.g., ACMG/AMP evidence codes PP3, PM2, PP1) were applied qualitatively to contextualize rarity, segregation, and in silico evidence, without assigning formal clinical classifications or revising categorical pathogenicity labels [7,9,11,12]. In silico tools are well suited for prioritizing variants, nominating pathways, and integrating heterogeneous datasets, but they cannot by themselves determine effect size, directionality, or tissue‑specific penetrance of individual alleles; accordingly, the TRIO-ras homolog family member A (RHOA)-guanine nucleotide binding protein subunit beta 1 (GNB1)-CACNA2D2 axis proposed here should be regarded as an experimentally testable circuit that organizes existing genetic and bioinformatic evidence, rather than as proof of a fully defined signaling pathway in human teeth [7,9,11,12].

Protein-protein interaction and pathway analysis

Protein-protein interaction networks were constructed using the Search Tool for the Retrieval of Interacting Genes/Proteins (STRING; version 11.5), which aggregates multiple functional association channels (experiments, curated databases, co‑expression, genomic context, and text mining) into a unified confidence score rather than directly quantifying physical binding [7,9,11,12]. In practice, we focused on edges with contributions from the “experiments” and “databases” channels, which aggregate biochemical and genetic interaction data as well as curated complex and pathway resources, and we required a highest-confidence combined score of at least 0.9 (top ~1% of associations). We manually examined the underlying evidence for edges connecting TRIO, RHOA, GNB1, and CACNA2D2 and did not use text mining or purely predictive signals as sole justification for including any edge in the proposed TRIO-RHOA-GNB1-CACNA2D2 axis [7,11,12]. Networks were visualized in the Cytoscape network visualization platform (Cytoscape), and centrality and enrichment metrics were interpreted descriptively as indicators of node positioning within functional neighborhoods rather than as measures of mechanistic causality [13].

Statistical analysis

Quantitative analyses in this study were primarily descriptive. For the conservation analysis, percent identity between human TRIO or CACNA2D2 and each vertebrate orthologue was calculated for both local windows and full‑length proteins, and values were summarized numerically without formal hypothesis testing. For network‑based pathway enrichment, over‑representation of Gene Ontology (GO) and Kyoto Encyclopedia of Genes and Genomes (KEGG) terms among first‑neighbor interactors was assessed using the default statistical framework implemented in the interaction database (hypergeometric testing with multiple‑testing correction), and only terms with adjusted p‑values below the predefined significance threshold were highlighted. Centrality measures (degree and betweenness) were reported as descriptive metrics to characterize node positioning within the local network rather than for inferential comparison [7,9,11,12].

## Results

Previously reported familial context

The trio Rho guanine nucleotide exchange factor (TRIO) c.8312C>T (p.Ser2771Leu) and calcium voltage‑gated channel auxiliary subunit alpha2delta 2 (CACNA2D2) c.284G>A (p.Arg95His; reference SNP identifier [rs] 149979955) variants examined here were originally identified in two Lebanese families with familial nonsyndromic tooth agenesis and were shown to co‑segregate with the dental phenotype in informative individuals in that prior clinical and whole‑exome sequencing study. The present analysis does not revisit detailed phenotypic or sequencing information, which has been published previously, but instead uses these variants as starting points for deeper mechanistic, structural, and network‑level interpretation.

Domain mapping places both variants in functional protein regions

Domain annotation showed that CACNA2D2 p.Arg95His lies in the extracellular N‑terminal segment immediately upstream of the von Willebrand factor type A (VWA) domain, a module that is central to α2δ‑subunit biology and implicated in channel trafficking and metal‑dependent functional regulation [6,7,11,12]. Protein‑domain annotation likewise placed the TRIO c.8312C>T (p.Ser2771Leu) substitution within an immunoglobulin I‑set domain directly adjacent to the C‑terminal kinase‑like region [6,7,9,11,12]. Table 1 summarizes transcript identifiers, Human Genome Variation Society (HGVS) nomenclature, and domain context for both variants to provide a concise reference for subsequent conservation, structural, and network analyses [6,7,9,11,12].

**Table 1:** Basic annotation and domain context for trio Rho guanine nucleotide exchange factor (TRIO) and calcium voltage gated channel auxiliary subunit alpha2delta 2 (CACNA2D2) variants were analyzed in this study. This table lists transcript identifiers, coding‑ and protein‑level nomenclature, and domain context for the TRIO p.Ser2771Leu and CACNA2D2 p.Arg95His missense variants, highlighting their localization within an immunoglobulin I‑set domain and an N‑terminal segment proximal to the von Willebrand factor type A (VWA) domain, respectively.

Gene	Transcript ID*	cDNA change	Protein change	Domain location	Nearby functional domains
TRIO	NM_007118.4 (ENST00000344204.9)	c.8312C>T	p.Ser2771Leu	Immunoglobulin I‑set domain	Directly adjacent to the C‑terminal kinase‑like region
CACNA2D2	NM_006030.4 (canonical Ensembl transcript for ENSG00000007402)	c.284G>A	p.Arg95His	N‑terminal segment immediately upstream of the VWA domain	Near the von Willebrand factor type A (VWA) domain involved in metal‑ion binding and modulation of voltage‑gated calcium‑channel activity

Residue conservation supports evolutionary constraint

**Figure 1:**
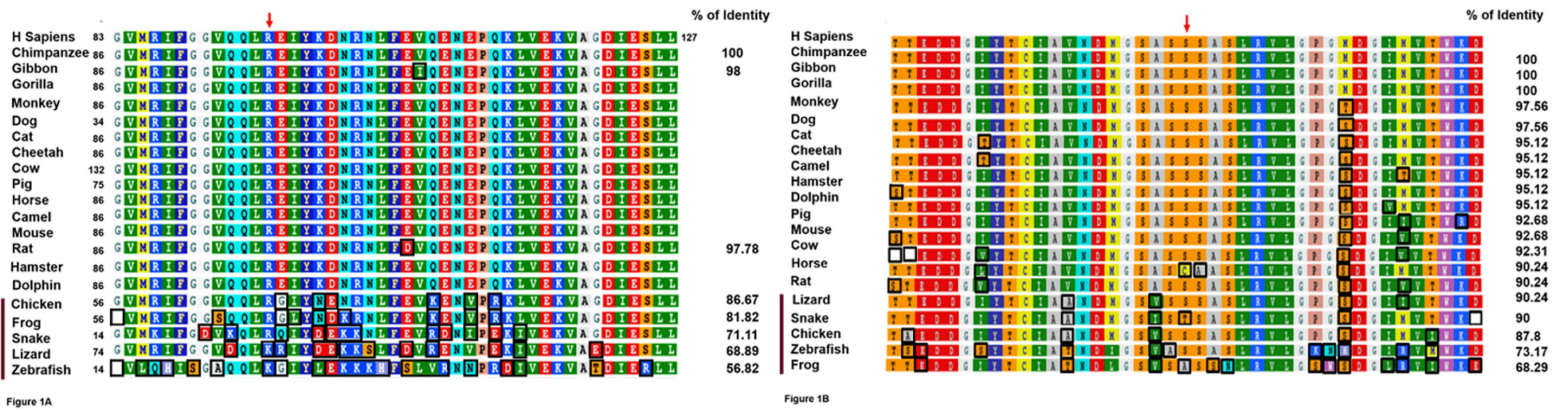
Evolutionary conservation around the calcium voltage-gated channel auxiliary subunit alpha2delta 2 (CACNA2D2) p.Arg95His and trio Rho guanine nucleotide exchange factor (TRIO) p.Ser2771Leu variant sites. (A) Multiple‑sequence alignment of CACNA2D2 orthologues from representative mammalian and non‑mammalian vertebrate species spanning residues 83–127, illustrating complete conservation of Arg95 in all examined mammals and high local sequence identity across vertebrates. (B) Multiple‑sequence alignment of TRIO orthologues across residues 2750–2790, showing preservation of Ser2771 in all mammalian orthologues and marked conservation of the surrounding region. Protein sequences were retrieved using the Basic Local Alignment Search Tool (BLAST), HomoloGene, and National Center for Biotechnology Information (NCBI) orthology resources, aligned with the Constraint‑based Multiple Alignment Tool (COBALT), and visualized with the Multiple Sequence Alignment Viewer using an amino acid property‑based coloring scheme. The final multi‑panel figure layout was assembled in Microsoft PowerPoint (Microsoft Corporation, Redmond, WA, USA).

Cross‑species alignment of the CACNA2D2 local window showed that Arg95 was identical in all 15 mammalian orthologues examined and remained preserved in most non‑mammalian vertebrates in the alignment panel. Identity across the 83-127 window ranged from complete conservation in closely related mammals to substantially lower similarity in distant vertebrates, indicating that Arg95 lies within a constrained local segment rather than an unconstrained variable loop [6,7,11,12]. The conservation patterns around Arg95 in calcium voltage‑gated channel auxiliary subunit alpha2delta 2 (CACNA2D2) and Ser2771 in trio Rho guanine nucleotide exchange factor (TRIO) are illustrated in the multi‑species alignments shown in Figures 1A, 1B, respectively, which highlight the preserved residues and local identity percentages across representative mammalian and non‑mammalian vertebrates.

A parallel analysis of trio Rho guanine nucleotide exchange factor (TRIO) showed that Ser2771 is likewise conserved in all examined mammalian orthologs and retained in most non‑mammalian vertebrates. Across the 2750-2790 window, local sequence identity remained sufficiently high to indicate that Ser2771 lies within a conserved structural segment, consistent with a functional constraint on this region of the protein [7,9,11,12].

Taken together, these conservation patterns make it more plausible that substitution at either site could have biological consequences, especially when interpreted in the context of their domain localization and the familial segregation data [1-3,6,9,12].

Structural inspection suggests local perturbation potential

Inspection of available structural models’ places calcium voltage‑gated channel auxiliary subunit alpha2delta 2 (CACNA2D2) Arg95 is immediately upstream of the von Willebrand factor type A (VWA) domain in a region that is likely to influence the folding and spatial presentation of this module [1-3,7]. Replacing arginine with histidine at this position could modify local electrostatics, side‑chain contacts, or domain‑proximal packing, and thereby alter how the neighboring VWA‑containing region is stabilized or displayed in the extracellular space [7,11,12]. For trio Rho guanine nucleotide exchange factor (TRIO), Ser2771 lies within the immunoglobulin I‑set (I‑set) domain close to the kinase‑like region, and substitution of a conserved polar residue with a bulkier hydrophobic leucine may perturb local hydrogen‑bonding networks or packing within the folded domain, potentially affecting domain integrity or interaction interfaces [1-3,6,7].

These interpretations are qualitative and do not quantify the magnitude or direction of any stability change, but they support the view that both variants alter structurally meaningful sites where even subtle perturbations could modulate protein behavior [1-3,11,12]. To keep the analysis conservative and transparent, we restricted structural interpretation to qualitative inspection of experimentally derived templates and full‑length AlphaFold Protein Structure Database (AlphaFold) models, without applying free‑energy change estimators, interface‑disruption predictors, or position‑specific conservation scoring tools. Accordingly, the structural component of this study should be regarded as pointing to plausible local packing and electrostatic effects at the variant sites, rather than providing quantitatively benchmarked estimates of stability or binding energetics. Nevertheless, the placement of both residues within locally conserved sequence windows and annotated domains suggests that even modest structural perturbations at these positions are less likely to be functionally neutral than changes occurring in poorly conserved, unstructured regions [6,7,11,12].

Population rarity and computational support remain compatible with candidacy

In the original analysis, CACNA2D2 p.Arg95His (rs149979955) was observed at very low allele frequencies in the Genome Aggregation Database (gnomAD) and other population datasets, and although Clinical Variants (ClinVar) lists it as likely benign in a non‑dental context, its rarity remains compatible with consideration as a candidate allele for familial tooth agenesis when interpreted together with segregation and domain localization [6,7,9,11,12]. In contrast, the TRIO p.Ser2771Leu substitution has not been detected in available reference cohorts and is absent from ClinVar, supporting its classification as an ultra‑rare, possibly private variant in the studied families [1-3,11,12].

The combination of cross‑species conservation, very low or absent population allele frequencies, and convergent predictions from multiple in silico tools supports continued prioritization of both TRIO p.Ser2771Leu and CACNA2D2 p.Arg95His as hypothesis‑generating candidates rather than neutral polymorphisms. These features are summarized in Table 2 to enable side‑by‑side comparison between the two genes. Taken together, rarity, segregation, conservation, and computational prediction keep TRIO p.Ser2771Leu at the forefront as a strong candidate variant, whereas CACNA2D2 p.Arg95His remains a plausible but more cautious signal whose interpretation is clearly context‑dependent [1-3,11,12]. In practice, TRIO p.Ser2771Leu can be regarded as an ultra‑rare, segregating variant in a gene with compelling developmental plausibility and no conflicting clinical annotations, while CACNA2D2 p.Arg95His is best viewed as a very rare allele with supportive conservation and in silico data but low‑frequency observation in population datasets and a prior likely benign ClinVar annotation in an epilepsy setting [6,7,9,11,12]. Accordingly, we treat CACNA2D2 p.Arg95His as a more speculative, context‑dependent contributor, compatible with a potential modifier or oligogenic role in Family 2 rather than a stand‑alone, high‑confidence causal variant.

**Table 2:** Conservation, population frequency, and in silico pathogenicity evidence for trio Rho guanine nucleotide exchange factor (TRIO) and calcium voltage-gated channel auxiliary subunit alpha2delta 2 (CACNA2D2) variants. This table integrates residue conservation across 20 vertebrate orthologues, allele frequencies from large reference datasets, Clinical Variants (ClinVar) and other database annotations, and predictions from six missense‑effect tools—Sorting Intolerant From Tolerant (SIFT), Polymorphism Phenotyping version 2 (PolyPhen‑2), likelihood ratio test (LRT), MutationTaster, MutationAssessor, and Functional Analysis through Hidden Markov Models (FATHMM)—for TRIO p.Ser2771Leu and CACNA2D2 p.Arg95His, collectively supporting their interpretation as rare, conserved candidate variants in the setting of familial tooth agenesis.

Gene	Protein change	Conservation across 20 species	Population data (gnomAD / others)	ClinVar / database	In‑silico effect (6 tools)	Interpretation
TRIO	p.Ser2771Leu	Ser2771 identical in all 15 mammals; conserved in most of 5 non‑mammalian vertebrates; local window 2750–2790 shows 100–68.29% identity to human	Absent from gnomAD exomes (123,136 individuals) and genomes (15,496); not reported in ClinVar	Not reported in ClinVar; missense‑constrained gene	Predicted damaging by ≥3/6 tools (SIFT, PolyPhen‑2, LRT, MutationTaster, MutationAssessor, FATHMM)	Strong candidate variant co‑segregating with tooth agenesis in Family 1
CACNA2D2	p.Arg95His	Arg95 identical in all 15 mammals; conserved in most of 5 non‑mammalian vertebrates; local window 83–127 shows 100–56.82% identity to human	gnomAD exome AF 0.000446 (101 exomes, 3 genomes); TOPMed AF 0.000183; PAGE AF 0.00018; all very rare	Single ClinVar entry (likely benign in epilepsy context)	Predicted damaging by ≥3/6 tools (same panel)	Candidate variant co‑segregating with tooth agenesis in Family 2; overall evidence remains compatible with a variant of uncertain significance in this context

Network analysis supports a TRIO-RHOA-GNB1-CACNA2D2 axis

**Figure 2:**
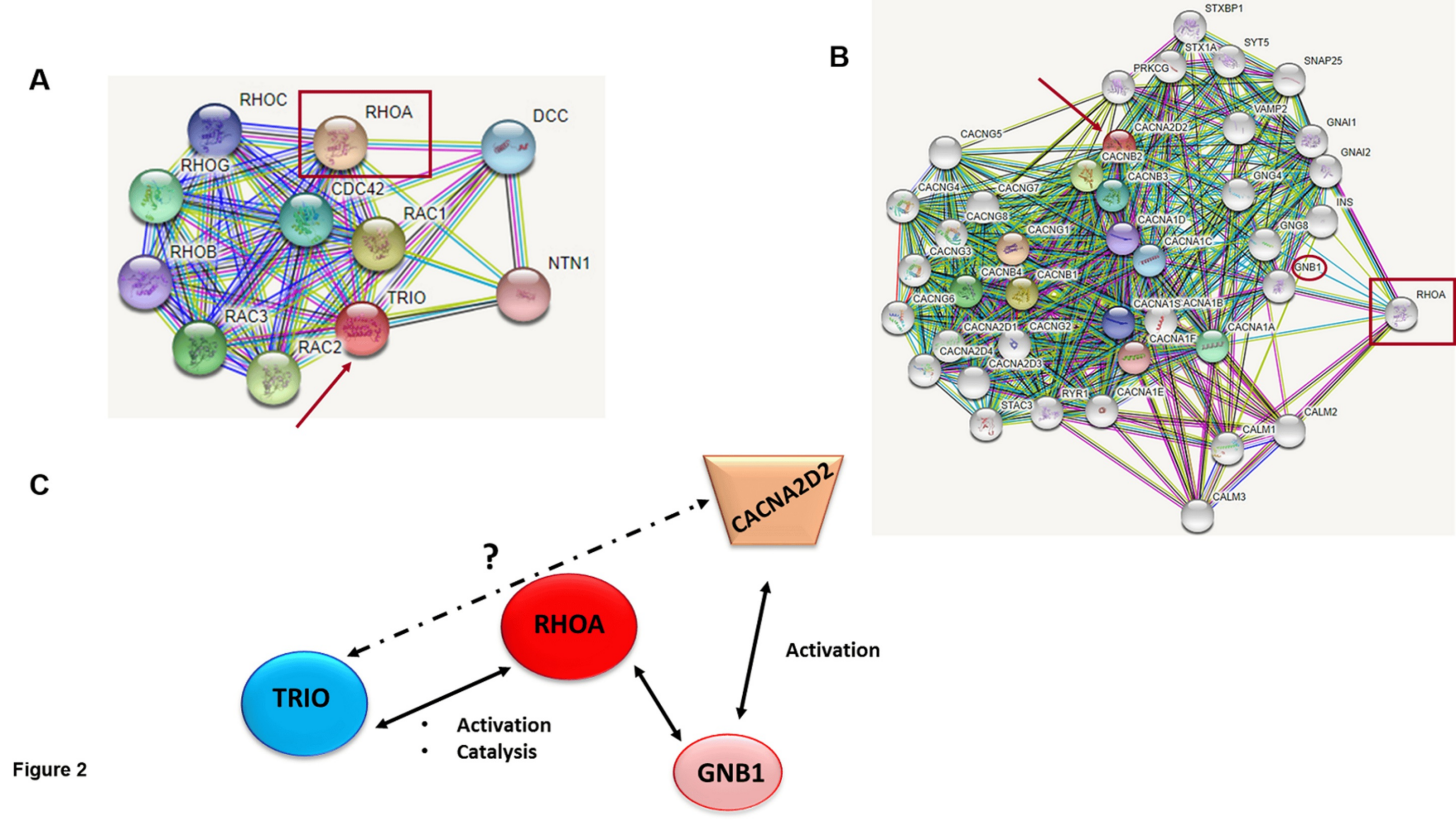
Proposed trio Rho guanine nucleotide exchange factor (TRIO)–ras homolog family member A (RHOA)–guanine nucleotide binding protein subunit beta 1 (GNB1)–calcium voltage gated channel auxiliary subunit alpha2delta 2 (CACNA2D2) signaling axis in tooth development. (A) Protein–protein interaction network illustrating the predicted functional link between TRIO and RHOA. (B) Network view showing the interaction between RHOA and the G protein β subunit GNB1 and the positioning of GNB1 within a broader calcium‑channel–related neighborhood. (C) Schematic representation of a putative TRIO–RHOA–GNB1–CACNA2D2 axis, consistent with a potential modulatory role of CACNA2D2 in coupling Rho guanosine triphosphatase (GTPase)–dependent cytoskeletal regulation to voltage‑gated calcium‑channel activity. In the context of the Lebanese pedigrees, TRIO is shown as a strong candidate variant, whereas CACNA2D2 is depicted as a more speculative contributor; the network is intended to illustrate convergent protein‑level pathways rather than equivalent variant‑level evidence. Nodes represent proteins, and edges represent curated or predicted interactions; line thickness reflects interaction confidence. Interaction networks were generated in the Cytoscape network visualization platform (version 3.10.3) from interaction data retrieved from the Search Tool for the Retrieval of Interacting Genes/Proteins (STRING) database (version 11.5) using a combined score threshold of ≥0.9, and final schematic layouts were assembled in Microsoft PowerPoint (Microsoft Corporation, Redmond, WA, USA).

High‑confidence Search Tool for the Retrieval of Interacting Genes/Proteins (STRING) analysis positioned trio Rho guanine nucleotide exchange factor (TRIO) in a direct functional neighborhood with Ras homolog family member A (RHOA), consistent with the established role of TRIO as a Rho guanine nucleotide exchange factor (Figure 2A). A second interaction neighborhood linked RHOA to guanine nucleotide binding protein subunit beta 1 (GNB1), and GNB1 in turn showed predicted associations with voltage‑gated calcium‑channel components that include calcium voltage‑gated channel auxiliary subunit alpha2delta 2 (CACNA2D2), thereby providing an indirect bridge between Rho guanosine triphosphatase (GTPase) signaling and calcium‑channel regulation (Figure 2B). The resulting interaction map delineates a TRIO-RHOA-GNB1-CACNA2D2 path that should be interpreted as a protein‑level scaffold, agnostic to the pathogenicity of any specific variant (Figure 2C), and Figure 2 as a whole illustrates this local network, highlighting the central positioning of these nodes and the enrichment of cytoskeletal, vesicle‑trafficking, calcium‑signaling, and mineralization terms among their immediate interactors [1-3,6,11,12].

Within this local interactome, trio Rho guanine nucleotide exchange factor (TRIO), ras homolog family member A (RHOA), guanine nucleotide binding protein subunit beta 1 (GNB1), and calcium voltage‑gated channel auxiliary subunit alpha2delta 2 (CACNA2D2) exhibited moderate‑to‑high degree and betweenness values relative to neighboring nodes, suggesting that they act as connectors linking cytoskeletal, signaling, and ion‑channel subnetworks. This configuration aligns with a model in which morphogenetic regulation and calcium‑dependent processes are coordinated rather than operating as isolated modules during odontogenesis.

Pathway enrichment of immediate network neighbors highlighted Gene Ontology (GO) and Kyoto Encyclopedia of Genes and Genomes (KEGG) terms related to cytoskeletal organization, vesicle trafficking, calcium signaling, and mineralization. Although these enrichments do not demonstrate that the specific variants disrupt these processes in vivo, they provide a coherent systems‑level framework in which the two candidate genes can converge biologically and support a testable TRIO-RHOA-GNB1-CACNA2D2 signaling axis in tooth development.

## Discussion

Taken together, this study should be viewed as a mechanistic, hypothesis‑generating exercise that refines where and how trio Rho guanine nucleotide exchange factor (TRIO) and calcium voltage‑gated channel auxiliary subunit alpha2delta 2 (CACNA2D2) might act in human odontogenesis, rather than as definitive evidence that these variants cause tooth agenesis. Within this framework, the network analysis functions primarily as an organizing layer that links several independent lines of evidence, including familial segregation, residue‑level conservation, domain localization, and structural modeling, into a coherent and testable hypothesis. Such secondary in silico analyses are increasingly valuable in dental genetics because they can extract additional biological insight from rare familial datasets, especially when recruiting new families or performing wet‑lab experiments is logistically difficult. Recent orthodontic cohort data have underscored the clinical burden of permanent tooth agenesis and its frequent association with additional dental anomalies, further motivating detailed mechanistic analyses of candidate variants [14]. The earlier clinical and whole‑exome sequencing study established the pedigrees, phenotypes, segregation patterns, and the presence of rare TRIO and CACNA2D2 missense variants in these families, whereas the present work was designed as a mechanistic extension focused on additional domain‑level, evolutionary, structural, and network‑based interrogation of those same variants. Nonetheless, the exclusively in silico secondary design and the absence of functional validation mean that the proposed TRIO-RHOA-GNB1-CACNA2D2 axis should be interpreted strictly as a limitation‑aware, hypothesis‑generating model rather than as evidence of causality.

A central observation is that both p.Ser2771Leu and p.Arg95His occur in structured, functionally annotated protein regions rather than in poorly characterized sequence space. For candidate variants in underexplored tooth-agenesis genes, such localization matters because it strengthens the argument that a rare substitution is occurring in a potentially interpretable functional context. In TRIO, placement within an immunoglobulin I-set (I-set) domain directly adjacent to a kinase-like region suggests possible effects on domain integrity or intermolecular interactions. In CACNA2D2, positioning immediately N-terminal to the von Willebrand factor type A (VWA) domain points to potential consequences for local architecture and α2δ-dependent channel biology, including channel trafficking or metal-dependent modulation [9-12].

The conservation analysis adds depth beyond a simple statement that the residues are conserved. By quantifying local identity across vertebrate panels and demonstrating preservation of Ser2771 and Arg95 across all examined mammals, the data support the inference that both positions lie under evolutionary constraint within locally conserved sequence windows [6,9-12]. This does not establish pathogenicity, but it narrows the interpretive space by making functional neutrality less biologically intuitive than it would be for poorly conserved sites and supports the view that substitutions at these positions merit mechanistic attention [9-12].

The most distinctive contribution of the present work is the network-based proposal of a TRIO-ras homolog family member A (RHOA)-guanine nucleotide binding protein subunit beta 1 (GNB1)-CACNA2D2 axis. This model is appealing because it links two apparently separate biological themes, namely Rho guanosine triphosphatase (GTPase)-driven cytoskeletal organization and calcium-channel-associated signaling, within a single framework relevant to tooth development [9-13]. Tooth morphogenesis requires coordinated cellular movement, polarity, and tissue organization, whereas dentin and enamel formation depend on regulated calcium handling and matrix mineralization. A pathway that couples these processes is therefore biologically plausible in principle, and the positioning of TRIO, RHOA, GNB1, and CACNA2D2 as connectors within an interaction neighborhood enriched for cytoskeletal regulation, vesicle trafficking, calcium signaling, and mineralization provides systems-level support for such coupling [9-13,15].

A key question is whether the TRIO-RHOA-GNB1-CACNA2D2 axis is plausibly embedded in ameloblast and odontoblast biology, rather than reflecting a generic cytoskeletal-calcium module [16-20]. Tooth-germ studies in humans and mice show that the cap-to-bell transition and subsequent crown formation are accompanied by coordinated differentiation of ameloblasts from inner enamel epithelium and odontoblasts from dental papilla mesenchyme, with polarized matrix secretion and mineralization as hallmark features [16-20]. Single-cell RNA sequencing of developing human teeth and human induced pluripotent stem cell (hiPSC)-derived odontoblasts further shows that ameloblast and odontoblast maturation is driven by dynamic fibroblast growth factor (FGF), bone morphogenetic protein (BMP), Wnt, epidermal growth factor (EGF), and Hedgehog signaling, with robust up-regulation of genes involved in cytoskeletal organization, vesicle trafficking, and calcium handling as these cells acquire their secretory phenotype [16-20]. In this context, a TRIO-centered Rho GTPase module and an α2δ-containing calcium-channel complex, such as CACNA2D2, provide a plausible mechanistic link between the polarity and motility programs that shape tooth-germ architecture and the calcium-dependent secretory and mineralization machinery characteristic of mature odontoblasts and ameloblasts [16-20].

The analysis also helps clarify the relative weight of evidence for the two variants. TRIO p.Ser2771Leu remains the stronger signal because it is absent from large reference datasets, lies in a conserved, annotated domain, and retains compelling segregation-based support from the original familial study. CACNA2D2 p.Arg95His is more equivocal: it is observed at very low frequency in population datasets and carries a prior Clinical Variants (ClinVar) interpretation as likely benign in a non-dental context, yet the combination of familial segregation, local and global conservation, domain location, and network position keeps it relevant as a potential contributor or modifier rather than allowing it to be dismissed outright [1-4,11,12]. This nuanced view aligns with emerging concepts of oligogenic and multilocus contributions to tooth agenesis, in which individual variants may modulate risk or expressivity without acting as sole deterministic causes [7,20-27].

At the same time, this hierarchy of evidence is crucial when considering how TRIO and CACNA2D2 are positioned on the proposed axis. TRIO p.Ser2771Leu occupies the center of the model as a high-priority candidate that is fully compatible with a monogenic contribution in Family 1. CACNA2D2 p.Arg95His, given its low but nonzero population frequency and its likely benign ClinVar annotation in a neurological setting, is more appropriately viewed as a plausible modifier allele whose contribution to tooth agenesis risk may be modest, context-dependent, or even ultimately neutral [1-4,7,12]. Thus, the shared axis reflects the convergent biology of the encoded proteins, not the equivalence of the underlying variants [11,20-27].

## Conclusions

This work suggests that previously reported trio Rho guanine nucleotide exchange factor (TRIO) and calcium voltage‑gated channel auxiliary subunit alpha2delta 2 (CACNA2D2) variants from Lebanese families with nonsyndromic tooth agenesis may participate in a shared mechanistic framework. Both substitutions occur in conserved, functionally annotated protein regions, remain compatible with candidate pathogenic relevance based on rarity and computational support, and can be placed within a high‑confidence interaction landscape linking TRIO, ras homolog family member A (RHOA), guanine nucleotide binding protein subunit beta 1 (GNB1), and CACNA2D2. The resulting ion channel-Rho guanosine triphosphatase (GTPase) model does not establish causality, but it offers a coherent, testable explanation for how disturbances in cytoskeletal control and calcium‑channel-associated signaling could converge during tooth development. In this sense, the study provides a mechanistic extension of the prior clinical and sequencing report, creating a clearer platform for targeted experimental validation in dental and craniofacial developmental systems. The ultimate impact of this work will depend on whether future functional studies corroborate or revise the proposed TRIO-RHOA-GNB1-CACNA2D2 axis, but by articulating some concrete, biologically informed hypotheses, it narrows the search space for those experiments. Within this framework, TRIO p.Ser2771Leu emerges as a strong candidate variant, whereas CACNA2D2 p.Arg95His is best regarded as a more tentative, hypothesis‑generating signal whose eventual classification may range from benign to a modest modifier, depending on future functional and genetic data.
